# Real-Time Performance Prediction in Long-Distance Trail Running: A Practical Model Based on Terrain Difficulty and Pacing Variability

**DOI:** 10.3390/sports13110385

**Published:** 2025-11-04

**Authors:** Héctor Gutiérrez, Eduardo Piedrafita, Pablo Jesús Bascuas, Irela Arbonés, César Berzosa, Ana Vanessa Bataller-Cervero

**Affiliations:** 1 Facultad de Ciencias de la Salud, Universidad San Jorge, Autovía A-23 Zaragoza-Huesca, km 299, Villanueva de Gállego, 50830 Zaragoza, Spain; hgutierrez@usj.es (H.G.); pbascuas@usj.es (P.J.B.); cberzosa@usj.es (C.B.); avbataller@usj.es (A.V.B.-C.); 2 Instituto Nacional de Educación Física de Cataluña (INEFC), Universidad de Lleida, Partida la Caparrella s/n, 25192 Lérida, Spain; marbones@gencat.cat

**Keywords:** trail running, endurance sports, pacing strategy, performance monitoring, fatigue management

## Abstract

Trail running is a demanding endurance sport where performance prediction models often rely on laboratory testing or pre-race data, limiting their practical application. This study presents a real-time predictive model for marathon and ultra-trail races, based on variables recorded during the race, including uphill/downhill pace-times, terrain difficulty coefficients, and partial rankings. A total of 947 runners from the ‘Trail Valle de Tena’ event (Spain) were analyzed to develop equations that estimate total race time using only the first third of the race. The model incorporates weighted time (WT_n_), pacing variability (WTV_n,n+2_), and checkpoint percentile rank (CPR_n_), showing strong predictive power (adjusted R^2^ > 0.95) across sexes and race modalities. These variables reflect the runner’s ability to both overcome elevation and maintain consistent pacing, offering insights into fatigue management and performance optimization. The model enables coaches and athletes to monitor race progression, adjust strategies in real time, and potentially reduce injury risk through better control of effort intensity. Unlike laboratory-based models, this approach is fully applicable in field conditions and does not require prior testing. Further validation in similar endurance events is recommended to confirm its utility as a practical tool for training and competition planning.

## 1. Introduction

In trail running, multiple factors influence performance; however, there is still no clear consensus on which are the most decisive [1,2]. Some classical models have proposed different variables as possible factors that could be related to the final performance in a single test or competition in this sports discipline. These include: physiological variables, such as maximal oxygen consumption (VO_2_max), ventilatory threshold (VT), running economy or muscle strength; sex; training load, indicated by average distance or average speed; and terrain characteristics, such as elevation gain, altitude or technical difficulty [3,4,5,6,7]. Nevertheless, these factors have not yet yielded models with the sufficient predictive power to completely and reliably predict performance in trail running races. Alternatively, some studies have suggested adapting predictive models from other contexts by conducting laboratory tests or collecting pre-competition data, although these approaches are often complex and impractical in real-world settings [8,9]. Currently, validated models have not been identified in scientific literature that can accurately predict trail runner performance.

To date, performance prediction models in long-distance races have primarily aimed to estimate total race time (TT) through equations applied to endurance continuous races across different sports disciplines. Keogh et al. identified 114 equations designed to explain the relationship between road-marathon TT and a range of independent variables [8]. Of these, 61 equations used variables collected outside the laboratory (such as anthropometric measurements, previous tests results, and training history), while the remaining 53 included variables measured directly in the laboratory, including VO_2_max and skinfolds thickness. 50 of the variables collected were independent, being the most frequently used: time in the previous race (35.0% of equations), average weekly training distance (24.6%), age (17.5%), VO_2_max (16.7%), number of previously completed marathon races (13.2%), average training race pace (13.2%), longest training run distance (11.4%), and total distance covered in the previous 8–9 weeks (10.5%). The predictive power of these models was assessed using the coefficient of determination (R^2^ = [0.10, 0.99]) and the standard error of estimate (SEE = [0.27, 27.40 min]). According to the authors, the main limitation of these models was not considering variables such as elevation gain, sex, or expected weather conditions on the race day to predict running performance. In the context of trail running, external variables (such as terrain variability or race-specific characteristics) appear to play a more critical role in determining performance outcomes, and may be essential for accurately predicting TT. However, a key limitation of these studies is their reliance on laboratory-based data, which may restrict their applicability in real-world trail running contexts.

In recent years, various models have been proposed to predict performance in trail running. Ehrström et al. developed a model to explain performance in a 27 km trail race, including variables such as VO_2_max, the percentage of VO_2_max corresponding to the VT, fatigue index, and running economy at both 0% and 10% slopes (R^2^ = 0.98) [3]. In line with these results, Scheer et al. conducted three laboratory tests to generate a predictive model in which the most remarkable variable to explain performance was running speed at VO_2_max, obtained through an inclined incremental running test (R^2^ = 0.68) [9]. Furthermore, this model’s explanatory power increased substantially when the TT from the same test in the previous year was included (R^2^ = 0.99). However, conducting laboratory tests to implement these predictive models is often challenging due to the limited availability of appropriate facilities. Moreover, these models often depend on data collected prior to the race, which limits their capacity to provide real-time insights during competition. Models that rely solely on data collected during the race may overcome the limitations associated with requiring pre-race or laboratory-based variables.

For this reason, alternative approaches to predict trail running performance without relying on laboratory tests have been developed. Fogliato et al. proposed a model with 26 variables and their interactions to estimate partial time at each kilometer point, TT, and the probability of withdrawal during the race [7]. These variables included runner’s sociodemographic data (e.g., age, sex, nationality) and information on previous races participation (e.g., number of races, difficulty level, withdrawals). Therefore, this model requires intermediate times during the race to predict runner’s TT, which offers a greater accuracy than models considering intermediate times as the only predictive variable. However, it still requires data collection on the same test day to make the prediction. A key limitation of this model is its dependence on a specific runners-database of tests conducted under standardized quality conditions. Consequently, athletes without prior participations in these tests or with insufficient race history cannot be effectively analyzed.

To sum up, creating a predictive model for trail running performance is a complex task, and a multivariate combination of variables is required. Therefore, to develop a model that is more applicable in real-world sports contexts (one that avoids the need for laboratory tests or pre-race data collection from the runner) would be more practical and efficient. In this study, publicly available data from two types of races within a trail running event were used as a reference to predict TT in trail races with similar characteristics. just considering the runners pace-partial times and their rankings at specific checkpoints. Thus, the aims of this study were: (1) to weight the runners pace-partial times in a specific race sector based on its relative difficulty compared to the entire race; (2) to examine the relationship between partial times and TT; (3) to analyze differences in these weighted times throughout the race and across groups defined by race modality, sex, and relative ranking in the sector; (4) to create a predictive model based on the analyzed variables and check its predictive capacity for TT in these races.

## 2. Materials and Methods

The ‘Trail Valle de Tena’ is an annual trail running event held in the Tena Valley (Panticosa, Huesca, Spain), featuring multiple race modalities. For the present study, a total of 947 official race times were analyzed. These times correspond to trail runners’ performances recorded in two race modalities (4K and 8K), who completed their respective races across three consecutive editions (2017–2019), and do not necessarily represent 947 unique participants, as some runners may competed in multiple editions and/or modalities. This period corresponds to the last three consecutive years in which the event was held without interruption prior to the COVID-19 pandemic. The 2020 edition was cancelled due to public health restrictions, and the immediately subsequent editions experienced certain changes as a consequence of this situation (e.g., race organization, track alteration, runners’ profiles and participation, or environmental conditions). Therefore, the 2017–2019 period was chosen to ensure consistency in race format and conditions, allowing for a more reliable longitudinal analysis.

The 4K race consisted of a total track distance (d) of 42 km, an accumulated positive slope (S^+^) of 3500 m, and an International Trail Running Association (ITRA) points of 77. In contrast, the 8K race covered 78 km with a S^+^ of 6900 m and an ITRA points of 147. The raw data were obtained directly from the event’s official website (https://trailvalledetena.com/ [last accessed on 29 March 2025]), where the information is publicly available. All data were anonymized prior to analysis by removing participants’ names and using only bib number as unique identifiers for statistical processing. As the data does not contain any sensitive information, approval from an ethics committee was not required for the conduct of this research.

Although the use of open-access data offers practical advantages, it may also present certain limitations depending on the type of study conducted (for example, the lack of demographic details about participants or the possibility of repeated participation across different race editions).

### 2.1. Total and Relative Race Difficulty

The variables total difficulty and relative difficulty were analyzed for both race modalities. The ITRA assigns points to each race to quantify the physical effort or difficulty involved in completing a specific trail running track. This is based on ITRA difficulty factor (IDF^+^), which incorporates both the total distance and the accumulated uphill-elevation gain (or positive slope), as indicated in Equation (1) [7].

(1) $${IDF}^{+}=d+\frac{S^{+}}{100}$$

**Equation (1).** IDF^+^: ITRA difficulty factor for accumulated uphill-elevation gain; d: total track distance (km); S^+^: accumulated positive slope (m).

As shown, ITRA currently evaluates race difficulty based on S^+^. Nevertheless, given that trail running tracks are typically non-circular, this study proposes also incorporating the downhill-elevation loss or accumulated negative slope (S^−^) into the assessment. Including both ascent and descent would provide a more comprehensive measure of overall race difficulty. To this end, a dual-difficulty coefficient is proposed, reflecting the IDF^+^ structure but also considering S^−^, as presented in Equation (2).

(2) $${IDF}^{-}=d+\frac{S^{-}}{100}$$

**Equation (2).** IDF^−^: ITRA difficulty factor for accumulated downhill-elevation loss; d: total track distance (km); S^−^: accumulated negative slope (m).

Considering both difficulty coefficients and using the previously described event database, the characteristics of each race analyzed are as follows:

- 4K: d = 42 km, S^+^ = 3500 m, S^−^ = 4000 m, IDF^+^ = 77, IDF^−^ = 82.
- 8K: d = 78 km, S^+^ = 6900 m, S^−^ = 6950 m, IDF^+^ = 147, IDF^−^ = 148.

Following this proposed model, the ITRA relative difficulty coefficient (IRDC_n_) would be defined as the difficulty of a specific section normalized by the total difficulty of the race. In a trail race, a section is understood as the partial segment of the total track between two specific checkpoints. For a section with a net positive elevation gain (i.e., more ascent than descent meters), Equation (3) would be applied. Conversely, for a section with a net negative elevation (i.e., more descent than ascent meters), Equation (4) would be applied.

(3) $$I{RDC}_{n}=\frac{{IDF}^{+n}}{{IDF}^{+}}$$

**Equation (3).** IRDC_n_: ITRA relative difficulty coefficient; IDF^+n^: ITRA difficulty factor calculated for a certain “n” section with a net positive slope; IDF^+^: ITRA difficulty factor for accumulated positive slope.

(4) $$I{RDC}_{n}=\frac{{IDF}^{-n}}{{IDF}^{-}}$$

**Equation (4).** IRDC_n_: ITRA relative difficulty coefficient; IDF^−n^: ITRA difficulty factor calculated for a certain “n” section with a net negative slope; IDF^−^: ITRA difficulty factor for accumulated negative slope.

Table 1 presents the overlapping sections of the 4K and 8K race modalities considered in this study. To distinguish them in the analysis, the sections were identified as follows: section 1 (s_1_), from the start line (considered as checkpoint 0 [cp_0_]) to Garmo Negro, the first shared checkpoint (cp_1_); section 2 (s_2_), from cp_1_ to Bachimaña, the second shared checkpoint (cp_2_); section 3 (s_3_), from cp_2_ to Tebarray, the third shared checkpoint (cp_3_); section 4 (s_4_), from cp_3_ to Respomuso, the fourth shared checkpoint (cp_4_); and section 5 (s_5_), from cp_4_ to Musales, the fifth shared checkpoint (cp_5_).

For each section, the table reports the corresponding difficulty factors (IDF^+^, IDF^−^) and relative difficulty coefficients (IRDC_n_), allowing for a comparative analysis of effort distribution across shared segments.

### 2.2. Weighted Time, Weighted Time-Variability and Relative Ranking

Due to the variable intensity inherent in trail running races, final performance may depend on two factors: the ability to traverse sections at the highest possible speed, and the capacity to maintain this intensity throughout the entire track.

In this study, the weighted time for a section *n* (WT_n_) is introduced as an indicator of a runner’s ability to complete a race partial-section at maximal speed. WT_n_ for a given section is defined as the time spent in completing that section (T_n_) divided by its corresponding IRDC_n_ value, as shown in Equation (5). T_n_ is recorded when the runner passes the second checkpoint delimiting that section. In this proposal, hour (h) was chosen as the time unit for expressing both T_n_ and WT_n_ variables.

(5) $${WT}_{n}=\frac{T_{n}}{I{RDC}_{n}}$$

**Equation (5).** WT_n_: weighted time for a “n” section (h); T_n_: time spent in completing a “n” section (h); IRDC_n_: ITRA relative difficulty coefficient for a “n” section.

If effort intensity were constant and the race just consisted of uphill (or downhill) segments, WT_n_ would correspond to the total race time (TT). However, in trail running, the effort intensity is variable throughout the track, making it necessary to account for potential fluctuations in speed. To conduct this, the weighted time-variability (WTV_n,n+2_) is defined (Equation (6)), where WT_n+2_ represents the weighted time of a given ascending section (or analogously, a descending section), and WT_n_ corresponds to the weighted time of the previous section of the same type (ascent or descent). As with the other previously mentioned time-related variables, WT_n+2_ is also expressed in hours.

It is worth noting that the subscript notations *n* and *n* + 2 do not imply that the sections are adjacent in the track sequence, but rather that they are consecutive in terms of terrain type. For example, in this five-section track where sections 1, 3 and 5 were net uphill, and sections 2 and 4 were net downhill, WTV_n,n+2_ reflects the variability between two successive uphill (or downhill) segments, skipping over the intervening section of opposite slope (e.g., WTV_1,3_ represents variability between the first and third uphill sections, skipping the downhill section in between). This formulation allows for the assessment of speed loss or gain across comparable terrain types as the race progresses.

(6) $${WTV}_{n,n+2}=\frac{{WT}_{n+2}}{{WT}_{n}}-1$$

**Equation (6).** WTV_n,n+2_: weighted time-variability, calculated as the relative change in weighted time between two consecutive sections of the same slope type (h); WT_n_: weighted time of the earlier section (h); WT_n+2_: weighted time of the later section (h).

Given the potential association between some of these variables and runners’ performance levels [10], the checkpoint percentile rank (CPR_n_) is also defined. This variable indicates the percentile position of each runner based on their time in the common section of the race (4K/8K), regardless of race modality, when passing the checkpoint delimiting the end of the section.

### 2.3. Data Analysis

WT_n_, WTV_n,n+2_ and CPR_n_ were calculated under three different conditions (Table 2).

The mean and standard deviation (SD) were calculated for TT, WT_n_, and WTV_n,n+2_ based on three dichotomous grouping variables: race (4K, n = 764; 8K, n = 183), sex (female, n = 76; male, n = 871), and CPR_n_ quartile [11,12] (Q1, n = 237, Q2–Q4, n = 710).

Most variables did not follow a normal distribution according to the Kolmogorov–Smirnov test; therefore, all statistical analysis were conducted using the bootstrap method with 1000 simple random resamples [13].

Pearson’s correlation coefficient (*r*) was used to assess linear relationships between TT, T_n_, and WT_n_.

Paired-sample *t*-tests were conducted to analyze intra-group differences across race conditions (net positive slope: WT_1_–WT_3_, WT_3_–WT_5_; net negative slope: WT_2_–WT_4_). Independent samples *t*-tests were used to examine inter-group differences (race modality, sex, and rank_n_ quartile) for WT_n_ and WTV_n,n+2_. Cohen’s *d* was calculated to determine effect sizes for these comparisons [14].

Multiple linear regressions were performed using the “enter” method. TT was considered as the dependent variable, while WT_n_, WTV_n,n+2_ and CPR_n_ were included as independent variables for both race conditions. Entry and removal criteria were set at *p* > 0.05 and *p* > 0.10, respectively. Linearity and independence of residuals were assessed using the Durbin–Watson test, with values between 1 and 3 considered acceptable. Homoscedasticity was evaluated using a plot of standardized residuals vs. standardized predicted values.

Additionally, the Bland–Altman method was employed to assess systematic bias and random error in the prediction models, as well as to determine the limits of agreement (±1.96 SD) [15]. Residual normality was tested using the Shapiro–Wilk test. Multicollinearity was assessed via the variance inflation factor (VIF), with values above 10 indicating excessive multicollinearity. Cases with a Cook’s distance greater than 1 were identified as influential, and residuals exceeding ±3 SD were considered outliers. Both types of cases were excluded from the final analysis.

All statistical tests were conducted with a significance level of *p* < 0.05. Post hoc statistical power (1-β) was calculated to determine the adequacy of the sample size based on the observed effect sizes (*r* and *d*).

The statistical analyses were performed using SPSS software, version 30 (Chicago, IL, USA). Graphical representations of the figures were created using Microsoft Excel, as it provided clearer and more precise visual outputs.

## 3. Results

The means (±SD) TT for all runners according to the race modality were 9.98 ± 1.70 h (in 4K) and 18.35 ± 3.24 h (in 8K). Table 3 presents the total time (TT) for the race in both modalities (4K and 8K), disaggregated by sex, along with the corresponding number of participants.

TT and WT_n_ showed stronger linear correlations (WT_1_: *r* = 0.962; WT_2_: *r* = 0.974; WT_3_: *r* = 0.976; WT_4_: *r* = 0.973; WT_5_: *r* = 0.944) than TT and T_n_ (T_1_: *r* = 0.821; T_2_: *r* = 0.682; T_3_: *r* = 0.713; T_4_: *r* = 0.616; T_5_: *r* = 0.476). All correlations were statistically significant (*p* < 0.001) with statistical power (1-β) > 0.80. These strong correlations suggest that WT_n_ is a more robust predictor of total race time than T_n_, likely because WT_n_ accounts for the relative difficulty of each terrain segment, integrating both distance and elevation gain into the performance assessment.

WT_n_ and WTV_n,n+2_ for each terrain type segment (ascent #1, descent and ascent #2) are presented in Table 4. In the first (ascent #1) and second (descent) segments, WT_n_ showed statistically significant (*p* < 0.05), high-powered (1-β > 0.80) changes with large effect sizes (*d* < −0.80) across all grouping variables (entire sample, race modality, sex, and quartile) in intra-group comparisons. This indicates that the estimated TT, adjusted for the time spent in each segment and its relative difficulty, increased between an ascent (or descent) and the subsequent segment with a similar net slope. However, this pattern was not observed in the third terrain type segment (ascent #2), where effect sizes were medium (0.50 < *d* < 0.80 for the 8K and female groups), small (0.20 < *d* < 0.50 for the entire sample, male, and Q2–4 groups), or trivial (0.00 < *d* < 0.20 for the 4K and Q1 groups). Moreover, the observed effect in this segment indicated a decrease in WT_n_ (*d* > 0.00) suggesting a reduction in weighted time between sections 3 and 5 (opposite to the trend observed in the first two sections).

Regarding intra-group differences, all WT_n_ values showed statistically significant and high-powered differences for the race and quartile grouping variables with medium effect sizes (WT_1_, WT_3_, WT_5_ in the quartile comparison) and large effect sizes in the remaining comparisons. However, no significant differences were found in WT_n_ based on sex (−0.20 < *d* < 0.20; *p* > 0.05; 1-β < 0.80).

For WTV_n,n+2_, statistically significant and high-powered differences were observed across race groups, with a medium effect size in ascent #1 (*d* = 0.54), small in descent (*d* = 0.45), and large in ascent #2 (*d* = 0.89). The direction of the effect indicated greater WTV_n,n+2_ in the 4K race, suggesting higher pace variability in this group and more consistent pacing in the 8K race. For the quartile grouping variable, WTV_n,n+2_ differences were small in ascent #1 (*d* = −0.33), medium in descent (*d* = 0.64), and trivial in ascent #2 (*d* = 0.14). These results suggest that runners in Q1 exhibited lower variability in weighted time during the first ascent and greater variability during the descent compared to the rest of the participants. No significant differences in WTV_n,n+2_ were found based on sex (−0.20 < *d* < 0.20; *p* > 0.05; 1-β < 0.80).

**Figure 1:**
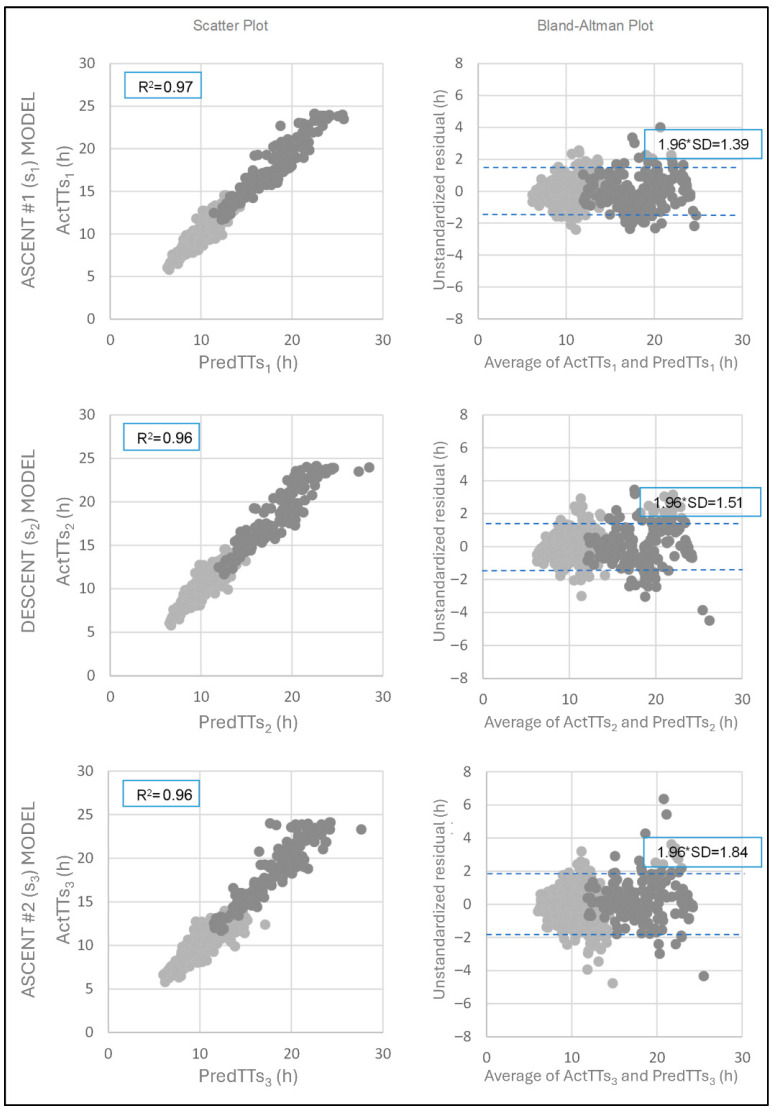
Scatter plots and Bland–Altman plots for the three multiple linear regression models corresponding to each terrain type segment: ascent #1 (section 1 [s_1_]); descent (section 2 [s_2_]); ascent #2 (section 3 [s_3_]). The scatter plots (left column) show the relationship between actual total race time and predicted total race time, with adjusted R^2^ values indicating strong model fit. The Bland–Altman plots (right column) display the agreement between predicted and actual times, with mean differences and limits of agreement (±1.96 SD). Model colors: light grey (4K); dark grey (8K). s_n_: race section analyzed; ActTTs_n_: actual total race time in each section; PredTTs_n_: total race time predicted by the model in each section; R^2^: adjusted coefficient of determination; SD: standard deviation; *: multiplication sign for limits of agreement determination.

Table 5 presents the three multiple linear regression models for each terrain type segment. The adjusted R^2^ values indicate that at least 96% of the variance in TT is explained by the proposed models (R^2^ ascent #1 = 0.967; R^2^ descent = 0.959; R^2^ ascent #2 = 0.961). The corresponding scatter plots for these models are shown in Figure 1. All models were statistically significant (*p* < 0.001), and all variables included in the models had a *p* < 0.001, supporting their inclusion.

**Figure 2:**
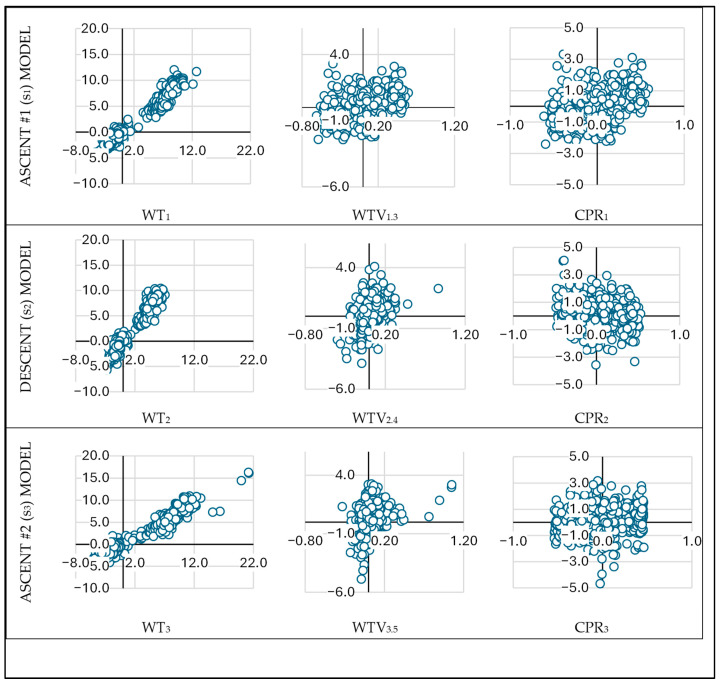
Partial standardized residuals (ordinate) plotted against standardized predicted values (abscissa) for three race terrain-segments (ascent #1 or section 1 [s_1_]; descent or section 2 [s_2_]; ascent #2 or section 3 [s_3_]) and key predictors. Each row corresponds to a different model, and each column represents a specific predictor: WT_n_ (weighted time in each section), WTV_n,n+2_ (weighted time-variability, calculated as the relative change in weighted time between two consecutive sections of the same terrain type), and CPR_n_ (checkpoint percentile rank: percentile position when passing at the ending checkpoint of a section s_n_). The plots illustrate varying degrees of linearity between residuals and predictors across models and terrain segments.

The Durbin–Watson test values fell within the acceptable range (1 < D-W < 3), confirming the assumptions of residual linearity and independence. In the partial standardized residual vs. standardized predicted value plots (Figure 2), residuals showed linear trends with respect to individual predictors: strong linearity for WT_n_, weak for WTV_n,n+2_, and very weak for CPR_n_ in the ascent segments (s_1_ and s_3_). However, Bland–Altman plots (Figure 2) showed residuals randomly distributed around the mean of TT and predicted time. Some values fell outside the ±1.96 SD limits, and residuals were not normally distributed, with outliers observed in Q1 and Q4. Despite this, no sport-related justification was found to exclude these cases, so they were retained in the analysis.

All variables had VIF values below 10, indicating no multicollinearity concerns.

## 4. Discussion

The primary aim of this study was to examine the relationship between TT (total race time) and WT_n_ (weighted time per section). The results demonstrated that WT_n_ exhibited stronger correlations with TT than raw section time (T_n_), suggesting that WT_n_ may serve as a more accurate predictor of final performance. This enhanced predictive capacity is likely due to the incorporation of relative difficulty (accounting for both distance and elevation gain) into the WT_n_ metric, thereby providing a more noticeable representation of the runner’s effort across different terrain segments (uphill/downhill).

This approach aligns with previous models that normalize section speed relative to average race speed to evaluate pacing strategies during competition [16,17]. Similarly to those models, WT_n_ reflects relative intensity by integrating the time spent in a section with its proportional contribution to the race’s total difficulty.

In support of the first aim, WT_n_ values for each analyzed section showed a very strong linear relationship (*r* > 0.950) with the final race time, underscoring its utility as a performance indicator.

The second aim focused on analyzing WT_n_ differences across race sections and between participant variable groups. WT_n_ increased between sections during ascent #1 and descent, but remained stable or decreased during ascent #2. These variations suggest that WT_n_ is sensitive to terrain-specific demands and may reflect the influence of technical factors (surface type [18], slope [19,20]), environmental conditions (temperature and humidity [16,21,22]), and accumulated fatigue [23]. The reduced magnitude of WT_n_ differences in later segments may indicate a stabilization of pace as the race progresses, consistent with prior findings of greater speed loss in early race stages [17]. These results are consistent with previous research indicating that longer race distances tend to promote more stable pacing strategies, as athletes adopt energy-conserving approaches to manage fatigue over extended durations. Studies have shown that pacing variability decreases as race length increases, reflecting a shift toward more regulated effort distribution in ultra-endurance events [24,25,26]. However, the absence of a significant correlation between overall performance and descriptors of pacing in trail ultramarathons with hilly terrain suggests that pacing behavior in these events may differ from other types of running competitions, highlighting the influence of environmental and course-specific factors on pacing dynamics [17].

WT_n_ also varied significantly across race modality and performance quartiles, with faster times observed in the 4K group and among top-performing runners (Q1). Interestingly, no significant differences in WT_n_ were found between sexes, suggesting that male and female runners exhibit similar pacing behavior in this type of trail competition. This finding is consistent with previous research indicating reduced sex-based performance differences in longer-duration events [27,28,29]. Furthermore, no significant sex-based differences were observed in WTV_n,n+2_, the metric representing variability in weighted time between consecutive segments of similar slope.

Regarding WTV_n,n+2_, greater pace variability was observed in the 4K group, while the 8K group maintained more consistent pacing. This may reflect the higher relative intensity and shorter duration of the 4K race. Additionally, Q1 runners exhibited lower WTV_n,n+2_ during the first ascent and higher variability during the descent, possibly due to superior strength and technical skills that enable efficient uphill pacing and faster downhill running [30,31]. These findings support the inclusion of WTV_n,n+2_ in predictive models of performance, as it captures meaningful differences in pacing strategies across performance levels.

The third aim involved developing multiple linear regression models incorporating WT_n_, WTV_n,n+2_, and CPR_n_ (checkpoint percentile rank) to predict TT. The models for each terrain segment (ascent #1, descent, ascent #2) demonstrated high predictive power (adjusted R^2^ > 0.95), comparable to previous models in endurance running literature [3,8,9]. All three predictors were statistically significant (*p* < 0.001), with WT_n_ contributing the most explanatory power (β = 0.952–1.031), followed by WTV_n,n+2_ (β = 0.079–0.152) and CPR_n_ (β = −0.033–0.109). Residual analysis revealed non-normality and increasing variance with race distance and runner position, as illustrated in Figure 1 and Figure 2.

From an applied perspective, these findings suggest that: (1) the ability to efficiently overcome elevation and distance (WT_n_) is the strongest predictor of performance; (2) maintaining consistent pacing (low WTV_n,n+2_) contributes to performance, although to a lesser extent; and (3) WTV_n,n+2_ offers predictive value across sexes and terrain types.

Compared to laboratory-based models [3,9], the proposed model offers practical advantages by relying solely on in-race data, eliminating the need for costly and less accessible testing facilities. However, it requires data from the same race edition, limiting its use for pre-race predictions. Future studies should validate this approach in races with varying characteristics (e.g., elevation, distance, technical difficulty). Despite potential differences in model coefficients, we hypothesize that WT_n_, WTV_n,n+2_, and CPR_n_ (calculated after completing approximately 30% of the race, including initial uphill and downhill sections) will consistently demonstrate high predictive capacity.

Therefore, considering the regression model for ascent #1, Equations (7) and (8), have been derived. Both equations can be used to estimate final race time at the Tebarray checkpoint (cp_3_) in the 4K and 8K races of the ‘Trail Valle de Tena’ event. These equations incorporate section times (T_1_ and T_2_) and CPR_1_, enabling real-time performance forecasting and strategic decision-making.

(7) $${TT}_{4K}=\left(0.914\times \frac{T_{1}}{0.27}\right)+\left(4.993\times \left[\frac{\frac{T_{3}}{0.16}}{\frac{T_{1}}{0.27}}-1\right]\right)+\left(1.468\times \frac{{CPR}_{1}}{n}\right)+0.939$$

**Equation (7).** TT_4K_: predicted total race time (h) for the 4K race modality; T_1_: time spent in section 1 (h); T_3_: time spent in section 3 (h); CPR_1_: checkpoint percentile rank at cp_1_ (ending checkpoint at section 1); *n*: total number of participants. This equation estimates TT at checkpoint cp_3_ using in-race data from the first ascent and descent segments.

(8) $${TT}_{8K}=\left(0.914\times \frac{T_{1}}{0.14}\right)+\left(4.993\times \left[\frac{\frac{T_{3}}{0.08}}{\frac{T_{1}}{0.14}}-1\right]\right)+\left(1.468\times \frac{{CPR}_{1}}{n}\right)+0.939$$

**Equation (8).** TT_8K_: predicted total race time (h) for the 8K race modality; T_1_: time spent in section 1 (h); T_3_: time spent in section 3 (h); CPR_1_: checkpoint percentile rank at cp_1_ (ending checkpoint at section 1); *n*: total number of participants. This equation estimates TT at checkpoint cp_3_ using in-race data from the first ascent and descent segments.

### 4.1. Practical Applications

To enhance the practical utility of this model, we propose several considerations for trail running coaches: first, they should recognize that final race time is largely determined by the runner’s ability to generate and sustain high uphill speed; second, training programs should therefore target both uphill performance and fatigue resistance; third, race plans should account for progressive speed loss during ascents, even among elite runners. Finally, field tests replicating race demands (such as vertical kilometer efforts and repeated ascents) may serve as valuable tools for monitoring performance and informing training interventions.

Moreover, the variables proposed in this study (WT_n_, WTV_n,n+2_, and CPR_n_) offer practical value as accessible, non-laboratory-based predictors. Their use enables athletes and coaches to monitor race progression and adjust pacing strategies in real time, without the need for prior testing or specialized equipment.

### 4.2. Limitations and Future Research Directions

While the predictive model developed in this study demonstrates strong accuracy and practical applicability within the context of the ‘Trail Valle de Tena’ event, certain considerations should be taken into account when interpreting its generalizability. The model is based on data from a single competition (2017–2019 editions), which provided a consistent and well-documented framework for analysis. Although this enhances internal validity, future studies should explore its applicability to other races with different terrain profiles, distances, and organizational formats.

Additionally, the female subgroup in the sample was relatively small, particularly in the 8K modality. While the model showed consistent predictive performance across sexes, further validation with larger female cohorts would strengthen its robustness in sex-based comparisons.

The Bland–Altman analysis revealed a high level of agreement between predicted and actual race times, although a small number of outliers were observed and residuals were not normally distributed. These findings do not undermine the model’s predictive capacity but highlight areas for refinement in future iterations.

Finally, the model relies on in-race data (e.g., section times and checkpoint rankings), which limits its use for pre-race predictions. However, this design choice aligns with the study’s objective: to provide a real-time, field-based tool for monitoring pacing and forecasting performance during competition.

Future research should aim to replicate and adapt this approach in other endurance events, testing its performance across diverse terrain profiles, race distances, environmental conditions, and athlete subgroups (e.g., elite vs. recreational). Such efforts will help confirm the model’s versatility and position this study as a foundation for further work in trail running performance prediction.

## 5. Conclusions

In conclusion, WT_n_, WTV_n,n+2_, and CPR_n_ demonstrated strong predictive capacity for TT in trail running, specifically in the marathon and ultra-trail race modalities, due to their similar characteristics to the 4K and 8K races analyzed in the present study. The proposed model enables accurate performance estimation using only a portion of the race, without requiring laboratory testing, and appears valid for both male and female athletes.

## Figures and Tables

**Table 1:** Values of covered distances, positive/negative slopes and total/relative race-difficulty at the five sections overlapped in the 4K and 8K races of the ‘Trail Valle de Tena’ during the 2017–2019 editions.

Section	aD (km)		sD (km)	sS^+^ (m)	sS^−^ (m)	nS	IDF^+n^	IDF^−n^	IRDC_n_	
	4K	8K							4K	8K
s_1_ (cp_0_-cp_1_)	5.5	31.0	5.5	1430	−10	+	20.5		0.27	0.14
s_2_ (cp_1_-cp_2_)	11.0	36.5	5.5	205	−1150	−		17.0	0.21	0.11
s_3_ (cp_2_-cp_3_)	16.5	42.0	5.5	650	−80	+	12.0		0.16	0.08
s_4_ (cp_3_-cp_4_)	20.5	46.0	4.0	75	−680	−		11.0	0.13	0.07
s_5_ (cp_4_-cp_5_)	24.5	50.0	4.0	470	−75	+	9.0		0.12	0.06

s_n_: sections 1–5; cp_n_-cp_n+1_: consecutive checkpoints that define each section; aD: accumulated distance from the start to the last checkpoint of each section (km); sD: section-partial distance (km); sS^+^: section uphill-positive slope (m); sS^−^: section downhill-negative slope (m); nS: net slope (sign indicates whether the section has more uphill meters [+] or downhill meters [−]); IDF^+n^: ITRA difficulty factor calculated for a section with a net positive slope; IDF^−n^: ITRA difficulty factor calculated for a section with a net negative slope; IRDC_n_: ITRA relative difficulty coefficient for each section.

**Table 2:** Weighted time, weighted time-variability and checkpoint percentile rank across assessed terrain types (ascent/descent).

Terrain Type (Elevation Profile)	WTn		WTV_n,n+2_	CPR_n_
	4K	8K		
Ascent #1 (s1, s3)	${WT}_{1}=\frac{T_{1}}{0.27}$	${WT}_{1}=\frac{T_{1}}{0.14}$	${WTV}_{1,3}=\frac{WT_{3}}{{WT}_{1}}-1$	CPR_1_ (percentile position at cp_1_)
	${WT}_{3}=\frac{T_{3}}{0.16}$	${WT}_{3}=\frac{T_{3}}{0.08}$		
Descent (s2, s4)	$WT_{2}=\frac{T_{2}}{0.21}$	$WT_{2}=\frac{T_{2}}{0.11}$	${WTV}_{2,4}=\frac{{WT}_{4}}{{WT}_{2}}-1$	CPR_2_ (percentile position at cp_2_)
	$WT_{4}=\frac{T_{4}}{0.13}$	$WT_{4}=\frac{T_{4}}{0.07}$		
Ascent #2 (s3, s5)	${WT}_{3}=\frac{T_{3}}{0.16}$	${WT}_{3}=\frac{T_{3}}{0.08}$	${WTV}_{3,5}=\frac{{WT}_{5}}{WT_{3}}-1$	CPR_3_ (percentile position at cp_3_)
	$WT_{5}=\frac{T_{5}}{0.12}$	$WT_{5}=\frac{T_{5}}{0.06}$		

s_n_: race section; T_n_: time spent in each section; WT_n_: weighted time in each section; WTV_n,n+2_: weighted time-variability, calculated as the relative change in weighted time between two consecutive sections of the same slope type; CPR_n_: percentile position of each runner when passing at the checkpoint (cp_n_) delimiting the end of section s_n_.

**Table 3:** Total time in hours expressed in mean ± SD for both modalities (4k and 8K) by sex of the runners.

Sex	4K	8K
Males (*n* = 871)	9.94 ± 1.71 h (*n* = 698)	18.30 ± 3.26 h (*n* = 173)
Females (*n* = 76)	10.42 ± 1.58 h (*n* = 66)	19.08 ± 2.85 h (*n* = 10)
Overall sample (*n* = 947)	9.98 ± 1.70 h (*n* = 764)	18.35 ± 3.24 h (*n* = 183)

**Table 4:** Intra-group differences in WT_n_ and inter-groups differences in WT_n_ and WTV_n,n+2_ across the three race situations depending on terrain segment: ascent #1, descent, and ascent #2.

			Intra-Group			Mean ± SD	Inter-Group			Mean ± SD	Inter-Group			Mean ± SD	Inter-Group		
Segment	Variable	Group	*d*	*p*	1-β		*d*	*p*	1-β		*d*	*p*	1-β		*d*	*p*	1-β
**Ascent #1**						**WT_1_ (h)**				**WT_3_ (h)**				**WTV_1,3_ (h)**			
	Race	4K	−2.32 *	<0.001	>0.99	7.30 ± 1.02	−6.29 *	<0.001	>0.99	9.70 ± 1.77	−4.96 *	<0.001	>0.99	0.33 ± 0.12	0.54 *	<0.001	>0.99
		8K	−2.18 *	<0.001	>0.99	16.85 ± 2.75				21.23 ± 3.85				0.26 ± 0.11			
	Sex	Males	−1.83 *	<0.001	>0.99	9.16 ± 4.13	0.05	0.578	0.11	11.96 ± 5.19	0.07	0.472	0.14	0.31 ± 0.12	0.11	0.262	0.23
		Females	−2.33 *	<0.001	>0.99	8.94 ± 3.33				11.58 ± 4.16				0.30 ± 0.09			
	Quartile	Q1	−2.15 *	<0.001	>0.99	7.40 ± 2.99	−0.58 *	<0.001	>0.99	9.28 ± 3.65	−0.66 *	<0.001	>0.99	0.28 ± 0.10	−0.33 *	<0.001	>0.99
		Q2–4	−1.93 *	<0.001	>0.99	9.73 ± 4.21				12.81 ± 5.23				0.32 ± 0.13			
	Total		−1.86 *	<0.001	>0.99	9.14 ± 4.10				11.93 ± 5.11				0.31 ± 0.12			
**Descent**						**WT_2_ (h)**				**WT_4_ (h)**				**WTV_2,4_ (h)**			
	Race	4K	−2.33 *	<0.001	>0.99	5.57 ± 1.22	−3.83 *	<0.001	>0.99	6.42 ± 1.27	−4.06 *	<0.001	>0.99	0.16 ± 0.12	0.45 *	<0.001	>0.99
		8K	−0.89 *	<0.001	>0.99	11.60 ± 2.58				12.71 ± 2.38				0.11 ± 0.11			
	Sex	Males	−1.10 *	<0.001	>0.99	6.73 ± 2.89	−0.04	0.730	0.09	7.63 ± 2.95	−0.04	0.733	0.09	0.15 ± 0.12	−0.13	0.110	0.29
		Females	−1.66 *	<0.001	>0.99	6.83 ± 2.47				7.74 ± 2.69				0.14 ± 0.08			
	Quartile	Q1	−1.74 *	<0.001	>0.99	4.96 ± 1.72	−0.92 *	<0.001	>0.99	5.91 ± 2.01	−0.84 *	<0.001	>0.99	0.21 ± 0.11	0.64 *	<0.001	>0.99
		Q2–4	−1.00 *	<0.001	>0.99	7.42 ± 2.91				8.21 ± 2.96				0.13 ± 0.12			
	Total		−1.13 *	<0.001	>0.99	6.74 ± 2.86				7.73 ± 2.93				0.15 ± 0.12			
**Ascent #2**						**WT_3_ (h)**				**WT_5_ (h)**				**WTV_3,5_ (h)**			
	Race	4K	−0.02	0.649	0.09	9.70 ± 1.77	−4.96 *	<0.001	>0.99	9.71 ± 1.91	−3.73 *	<0.001	>0.99	0.00 ± 0.10	0.89 *	<0.001	>0.99
		8K	0.67 *	<0.001	>0.99	21.23 ± 3.85				19.05 ± 4.15				−0.10 ± 0.16			
	Sex	Males	0.20 *	<0.001	>0.99	11.96 ± 5.19	0.07	0.472	0.14	11.57 ± 4.51	0.14	0.190	0.32	−0.13 ± 0.12	0.28	0.002	0.76
		Females	0.53 *	<0.001	>0.99	11.58 ± 4.16				10.95 ± 3.68				−0.05 ± 0.08			
	Quartile	Q1	0.05	0.494	0.12	9.28 ± 3.65	−0.71 *	<0.001	>0.99	8.81 ± 3.03	−0.72 *	<0.001	>0.99	0.00 ± 0.15	0.14	0.121	0.59
		Q2–4	0.28 *	<0.001	>0.99	12.81 ± 5.23				12.43 ± 4.49				−0.02 ± 0.11			
	Total		0.21 *	<0.001	>0.99	11.93 ± 5.11				11.52 ± 4.45				−0.02 ± 0.12			

Segment: segment type depending on terrain slope; 4K: 4K race modality; 8K: 8K race modality; Q1: runners’ time ranked in quartile 1; Q2–4: runners’ time ranked in quartiles 2, 3 and 4; Total: overall sample within each segment; *d*: Cohen’s d effect size; Intra-group: significance level (*p*) and statistical power (1-β) for mean differences between related samples (*t* test); Inter-group: significance level (*p*) and statistical power (1-β) for mean differences between independent samples (*t* test); WT_n_: weighted time in each section; WTV_n,n+2_: weighted time-variability between two consecutive sections of the same slope type; * effect size with *p* < 0.05 and 1-β > 0.80.

**Table 5:** Multiple linear regression models for three race situations depending on terrain segment: ascent #1, descent, ascent #2.

Terrain Type Segment (Coinciding Race Section)	*r*	R^2^	adR^2^	SEE	*p*	Durbin Watson			B	B-SE	Beta	*p*	B		VIF
						LL95%	UL95%						LL95%	UL95%	
Ascent #1 (s_1_)	0.984	0.967	0.967	0.71	<0.001	1.104	1.444	**constant**	0.939	0.095		<0.001	0.754	1.135	
								**WT_1_**	0.914	0.009	0.952	<0.001	0.896	0.931	1.176
								**WTV_1,3_**	4.993	0.227	0.156	<0.001	4.548	5.444	1.079
								**CPR_1_**	1.468	0.086	0.109	<0.001	1.295	1.633	1.175
Descent (s_2_)	0.979	0.959	0.959	0.80	<0.001	1.005	1.316	**constant**	1.796	0.087		<0.001	1.613	1.613	
								**WT_2_**	1.411	0.015	1.031	<0.001	1.389	1.381	1.519
								**WTV_2,4_**	3.321	0.249	0.102	<0.001	2.856	2.849	1.215
								**CPR_2_**	−0.465	0.118	−0.033	<0.001	−0.686	−0.693	1.477
Ascent #2 (s_3_)	0.980	0.961	0.961	0.77	<0.001	1.021	1.373	**constant**	2.295	0.088		<0.001	2.159	2.431	
								**WT_3_**	0.746	0.009	0.976	<0.001	0.735	0.757	1.347
								**WTV_3,5_**	2.555	0.211	0.079	<0.001	2.122	2.989	1.138
								**CPR_3_**	0.888	0.090	0.066	<0.001	0.703	1.073	1.202

s_1_: race-section 1; s_2_: race-section 2; s_3_: race-section 3; *r*: correlation coefficient; R^2^: determination coefficient; adR^2^: adjusted determination coefficient; SEE: standard error of estimation; *p*: significance level; LL95%: lower limit for 95% confidence interval; UL95%: upper limit for 95% confidence interval; B: multiple linear regression coefficients for each variable; B-SE: B’standard error; Beta: standardized coefficients; VIF: variance inflation factor; WT_n_: weighted time in each section; WTV_n,n+2_: weighted time-variability between two consecutive sections of the same slope type; CPR_n_: checkpoint percentile rank.

## Data Availability

The data processed and analyzed for the development of this work were obtained from the official website of the event: https://trailvalledetena.com/ (last accessed on 29 March 2025). The original contributions presented in the study are included in the article; further inquiries can be directed to the corresponding author.

## Abbreviations

The following abbreviations are used in this manuscript:

VO_2_max	Maximal oxygen consumption
VT	Ventilatory threshold
TT	Total race time
SEE	Standard error of estimate
SD	Standard deviation
d	Total track distance
ITRA	International Trail Running Association
S^+^	Accumulated positive slope
IDF^+^	ITRA difficulty factor for accumulated uphill-elevation gain
S^-^	Accumulated negative slope
IDF^-^	ITRA difficulty factor for accumulated downhill-elevation loss
IRDC_n_	ITRA relative difficulty coefficient
IDF^+n^	ITRA difficulty factor calculated for a certain “n” section with a net positive slope
IDF^−n^	ITRA difficulty factor calculated for a certain “n” section with a net negative slope
s_n_	Race section
T_n_	Time spent in each section
cp_n_	Race checkpoint
4K	4K race modality
8K	8K race modality
Q1	Runners’ time ranked in quartile 1
Q2–4	Runners’ time ranked in quartiles 2, 3 and 4
WT_n_	Weighted time in each section
WTV_n,n+2_	Weighted time-variability between two consecutive sections of the same slope type
VIF	Variance inflation factor
CPR_n_	Checkpoint percentile rank
ActTTs_n_	Actual total race time in each section
PredTTs_n_	Total race time predicted by the model in each section
